# ID 345 - ATS e Inovação: a experiência em um hospital universitário no sertão de Pernambuco

**DOI:** 10.5327/2237-9622.2025.v34s1.345

**Published:** 2025-11-25

**Authors:** Gyllyandeson de Araújo Delmondes, Jairo Pessoa da Silva, Allissany de Castro Passos Reis, Emanuela Oliveira Spinola, Izabelle Silva de Araújo

**Keywords:** Avaliação de Tecnologia em Saúde, Sistema Único de Saúde, pesquisa, iniciação tecnológica

## Abstract

**Introdução:**

A limitação de recursos tende a ser um dos grandes desafios vivenciados pelos sistemas de saúde, sobretudo os públicos. No Brasil, o Sistema Único de Saúde (SUS) experencia o crescimento exponencial de demandas, bem como sua complexificação relacionada às condições de saúde, ao envelhecimento populacional, aos agravos de saúde externos, produtos dos determinantes sociais, econômicos, políticos e culturais. Por outro lado, o constante desenvolvimento científico-tecnológico em saúde exige da gestão o processo de tomada de decisão, a fim de atender, de forma mais eficiente, eficaz e efetiva, o direito à saúde, garantindo o acesso equitativo às tecnologias com qualidade e segurança. Para tanto, a Avaliação de Tecnologias em Saúde (ATS) assume grande relevância, pois apoia a tomada de decisão por meio de evidências científicas que respaldem a incorporação ou a desincorporação de uma tecnologia. Concernente à inovação tecnológica, a ATS estabelece uma relação intrínseca, não somente concernente ao Monitoramento do Horizonte Tecnológico (MHT), ou seja, seu ciclo de vida, mas também aos instrumentos e às ferramentas do processo de avaliação. Assim, em que medida a inovação tecnológica dos instrumentos e das ferramentas de ATS pode impactar os processos de avaliação? O objetivo deste resumo é apresentar a inovação tecnológica de um instrumento de ATS a partir da experiência na primeira edição do Programa de Iniciação Tecnológica (PIT) da Empresa Brasileira de Serviços Hospitalares (Ebserh) em um hospital universitário no sertão de Pernambuco. Destarte que o trabalho apresenta grande relevância científica e social a partir do aprimoramento da ATS. Além disso, faz-se necessário evidenciar a autenticidade e a originalidade do estudo, o potencial de inovação, sobretudo pelo desenvolvimento no âmbito da iniciação tecnológica.

**Método:**

Os estudantes realizaram revisão sistemática com meta-agregação das bases de dados PubMed, EMBASE, Cochrane e Instituto Joanna Brigss (JBI), com dois avaliadores e uso da plataforma Rayyan©, e posterior meta-agregação pelo software livre IRaMuTeQ. Essa análise visou examinar a estrutura e a organização da síntese textual, correlacionando-as aos domínios do modelo do JBI para Cuidados em Saúde Baseados em Evidências (CSBE).

**Resultados:**

O processo resultou no desenvolvimento da ferramenta denominada Karrankas Score para ATS, composta por 36 perguntas distribuídas em 5 domínios ("Saúde Global", "Geração de Evidências", "Síntese de Evidências", "Transferência de Evidências" e "Implementação de Evidências").

**Conclusão:**

A vivência da iniciação tecnológica favoreceu aos estudantes a aproximação com o universo da pesquisa, a inter-relação científica-tecnológica, para além do desenvolvimento de um produto tecnológico que visa a uma avaliação estruturada e abrangente das ATS, buscando estabelecer um padrão universal na análise de sua eficácia, segurança e viabilidade. O estudo ainda oportunizou a aproximação com ATS e desdobramentos: estruturação de grupo de pesquisa em práticas baseadas em evidências cadastrado no Conselho Nacional de Desenvolvimento Científico e Tecnológico (CNPq); novas pesquisas na área, inclusive nos Núcleos de Avaliação de Tecnologias Sociais (Nats) dos hospitais universitários da Rede Ebserh na Região Nordeste; e premiações em eventos científicos, para além da possibilidade de desenvolvimento de software, mas sobretudo o potencial de aplicabilidade e contribuição para o acesso da população às tecnologias em saúde por meio da ATS.

